# Evaluation of underreporting tuberculosis in Central Italy by means of record linkage

**DOI:** 10.1186/1471-2458-12-472

**Published:** 2012-06-21

**Authors:** Lorenza Melosini, Umberto Vetrano, Federico L Dente, Michele Cristofano, Mauro Giraldi, Luciano Gabbrielli, Federica Novelli, Ferruccio Aquilini, Laura Rindi, Francesco Menichetti, Giulia Freer, Pierluigi L Paggiaro

**Affiliations:** 1 Cardio-Thoracic and Vascular Department, Ospedale Cisanello, Via Paradisa 2, 56100 Pisa, Italy; 2 Infectious Diseases Unit, University Hospital, Pisa, Italy; 3 Medical Direction University Hospital, Pisa, Italy; 4 Micobacteriology Unit, University Hospital, Pisa, Italy

**Keywords:** TB –notifications

## Abstract

**Background:**

Tuberculosis (TB) surveillance systems have some pitfalls outside of a National Tuberculosis Program and lack of efficient surveillance hampers accurate epidemiological quantification of TB burden.

In the present study we assessed the quality of surveillance at the University Hospital in Pisa (UHP), Italy, and TB incidence rates over a ten year period (1999–2008).

**Methods:**

Assessment of underreporting was done by record-linkage from two sources: databases of TB diagnoses performed in the UHP and the Italian Infectious Disease Surveillance (IIDS) system. Two different databases were examined: a) TB diagnoses reported in the Hospital Discharge Records (HDR) from three Units of UHP (Respiratory Pathophysiology, Pulmonology and Infectious Diseases Units) (TB database A); b) TB diagnoses reported in HDR of all Units of UHP plus TB positive cases obtained by the Laboratory Register (LR) of UHP (TB database B). For the TB database A, the accuracy of TB diagnosis in HDR was assessed by direct examination of the Clinical Record Forms of the cases. For the TB database B, clinical and population data were described, as well as the trend of incidence and underreporting over 10 yrs.

**Results:**

In the first study 293 patients were found: 80 patients (27%) with a confirmed TB diagnosis were underreported, 39 of them were microbiologically confirmed. Underreporting was related to age (Reported *vs* Non Reported, mean age: 49.27 ± 20 *vs* 55 ± 19, p < 0.005 ), diagnosis (smear positive *vs* negative cases 18.7 vs 81.2%, p = 0.001), microbiological confirmation (49% *vs* 51%, p < 0.05), X-ray findings (cavitary *vs* non-cavitary cases: 12.5 *vs* 87.5%, p = 0.001) but not to nationality.

In the second study, 666 patients were found. Mean underreporting rate was 69.4% and decreased over time (68% in 1999, 48% in 2008). Newly diagnosed TB cases were also found to decrease in number whereas immigration rate increased. Underreporting was related to nationality (Immigrants *v*s Italians: 18% vs 68%, p < 0.001), diagnosis (microbiological confirmation: 25% *vs* 75%, p < 0.01), kind of hospital regimen (hospitalized patients *vs* Day Hospital: 70% *vs* 16%, p < 0.001), and position of TB code in the HDR (TB code in first position *vs* in the following position: 39,5% *vs* 45% p < 0.001).

**Conclusions:**

TB is underreported in Pisa, particularly in older patients and those without microbiological confirmation. The TB code in first position of HDR seems fairly accurate in confirming TB diagnosis.

## Background

Tuberculosis (TB) is a underestimated problem in low incidence countries [1], where several problems are associated with TB: increase in incidence and disease severity for immigrants, introduction of drug-resistant strains from high-incidence countries, and reduction in physicians’ expertise [2,3].

In Europe homogeneous surveillance system and criteria for reporting TB are still lacking [4,5] and underreporting is a well-recognized problem [6-9]. The lack of complete surveillance systems hampers meaningful epidemiological quantification of TB and represents a public health problem which may lead to an increase in the risk of disease transmission and drug resistance development (with a consequent impact on the cost of TB management).

In Italy, under-reporting TB ranges from 12% [10] to 37-54% in different areas [11,12]; in particular, little information is available about the underreporting rate in Tuscany and in Pisa [13], and there is no systematic collection of data related to treatment outcome. The cornerstone of preventive medicine in Italy is the Hygiene Office (a part of the Health Prevention Service) which should link data transmission from clinicians to epidemiological units, look for new cases in high-risk population, check/screen contacts, promote health initiatives, and exchange information locally and nationally. As described by Migliori et al [6], italian clinicians are required to report all new TB diagnoses directly to the preventive services [14,15]. Clinicians working in a hospital are required, instead, for both inpatients and outpatients, to send notification to the Health Direction of the Hospital, which includes these data in a local TB register and which send the notification to the local Hygiene Office. Most of the communication is in paper format, and the notification is finally sent to the national health authorities (Istituto Superiore di Sanità, leading technical and scientific institute of the Italian National Health Service). The database of the local Hygiene Office (Italian Infectious Disease Surveillance system, IIDS) includes all notifications of TB sent by the different health services (GPs, hospitals, TB laboratories). Local Hygiene Office should ask to clinicians information about the treatment and the outcome of TB, but answering is not mandatory.

The present study was aimed firstly at assessing the quality of the local surveillance system in the University Hospital of Pisa, Central Italy, and secondly at quantifying TB burden, its incidence trend and epidemiological changes during ten years (1999–2008). Then, two studies were done. In the first study, a record linkage was done between two sources: 1) all the Hospital Discharge Records (HDR) for inpatients where a diagnosis of TB was reported, obtained by the Units primarily involved in the TB management in the University Hospital of Pisa (Infectious Diseases, Pulmonology and Respiratory Pathophysiology Units)(TB database A) and 2) Italian Infectious Disease Surveillance (IIDS) system of the local Hygiene Office. We then computed the underreporting rate, as a percentage between the number of cases obtained from IIDS and the total number of TB cases where a correct TB diagnosis was done. In the second part of the study, we tried to evaluate the total number of TB cases which occurred in the University Hospital of Pisa (all Units) through the union of all databases where TB was detectable in the HDR obtained by all Units of the University Hospital in Pisa, and in the TB register of the Micobacteriology Unit (TB database B). In this larger database, we evaluated clinical, population, and microbiological features, and also the underreporting rate.

## Methods

The University Hospital of Pisa is located in Tuscany (Central Italy). It is a tertiary hospital, with a rate of 80.000 admissions/year, most of them coming from North Western Tuscany [16]. It is also a reference hospital for patients from all over Italy.

### First study: underreporting rate in the TB database A

**Figure 1:**
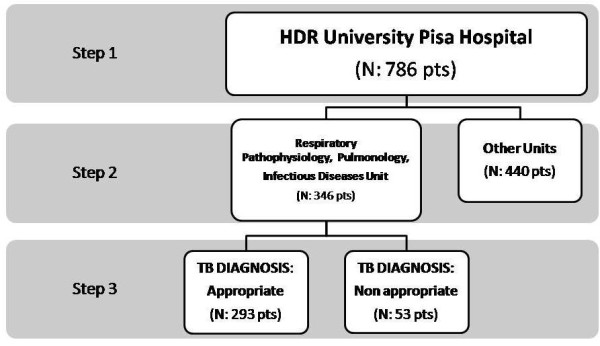
Data selection of patients according a step by step procedure (see text).

For the first part of the study we used two sources of data for record linkage, all related to the period between 01/01/1999 and 31/12/2008: HDR and IIDS. Figure 1 shows step by step the procedure used to select cases with a verified diagnosis of TB: from all HDR of the University Hospital of Pisa (Figure 1, step 1) we selected the cases discharged from tree Units only (Respiratory Pathophysiology, Pulmonology and Infectious Diseases) (Figure 1, step 2). A thorough examination of the Clinical Record Forms (CRF) of these patients was done: examination of these CRF allowed to confirm or not that the presence of a discharge diagnosis of TB in the HDR was appropriate (Figure 1, step 3) [14,15,17].We excluded outpatients observed in the University Hospital of Pisa for whom a HDR was not available and there was not the possibility to check for a correct TB diagnosis in the CRF. This database (TB database A) was crossed with the notification assessment (IIDS), in order to calculate the underreporting rate. 

Diagnosis of TB was considered appropriate when, from the direct examination of the CRF, it has been done according to the ATS/CDC 1999 criteria (17), therefore including both smear-positive or cultural-positive TB cases and TB cases with clinical diagnosis.

Discharge diagnoses of TB were classified according to the International Classification of Disease-IX codes. From all clinical record forms (CRF), demographic (age, gender, nationality, residence area), clinical (pulmonary and extra-pulmonary TB localization, HIV status, comorbidities) and microbiological data were collected. These observations were also crossed with the notification assessment (IIDS).

Furthermore, we verified the accuracy of having TB code in the first position of the HDR (main diagnosis) or in the following positions of the HDR of the University Hospital of Pisa, by crossing these data with the diagnosis of TB as assessed by the direct examination of the CRF of the three Units. The aim was to evaluate the accuracy of a TB code in the first position of the HDR.

### Second study: TB trend and underreporting rate over time in the TB database B

For the second part of the study, a second database was made by the union of two main databases of the University Hospital of Pisa, in which TB was detectable: HDR of all the Units of the University Hospital of Pisa and the laboratory TB register (LTR) of the Micobacteriology Unit including all cases with culture-confirmed *Mycobacterium tuberculosis* (MT). Duplicate cases were excluded from the analysis. For the patients recruited in this large TB database, demographic (age, gender, nationality, residence area), administrative (discharge Unit, TB code in HDR, hospitalized patients *vs* Day Hospital patients) and microbiological data were collected. For them a cross linkage was also done with notification register (IIDS).

Data regarding HDR in the archive of the University Hospital of Pisa were obtained from the Hospital server database, in agreement with existing privacy laws. The Authors were allowed to keep and analyze the data and produce anonymous reports. No informed consent was required according to the National law.

The observational study was approved from the Ethical Committee of the University Hospital of Pisa in the October 2008 (6584–2008).

### Statistical analysis

Cross linkage was made with Excel and Access programmes, between the TB databases A or B, and IIDS database. A “Find duplicates” query on multiple tables was created choosing the fields “surname”, “first name” and “date of birth”, each alone or in combination. The result set was then evaluated with a thorough manual check

Statistical analysis was done with SPSS. Quantitative variables were compared by Student’s *t* test for unpaired data. For gender, discharge Unit, nationality, residence area and other categorical variables, a chi square test was used. Positive Predictive Values (PPV) and Negative Predictive Values (NPV) were computed for TB in the first position of the HDR and the results of the examination of the hospital CRF (TB “confirmed” or “not confirmed”). A p-value of less than 0.05 was considered statistically significant.

## Results

### First study: underreporting rate in the TB database A

In the first part of the study, from a total of 786 HDR which included TB (any position) derived from all the University Hospital of Pisa (Figure 1, step 1), a total of 346 patients discharged from the Infectious Disease, Pulmonary and Respiratory Pathophysiology Units were found (Figure 1, step 2). Thorough direct examination of the CRF of these patients with a TB discharge code allowed to “confirm” (N = 293) or not (N = 53) active TB diagnosis (Figure 1, step 3).

For 293 patients with confirmed TB diagnosis, underreporting was 27% (Table 1). Notification rate was significantly higher for cavitary, smear positive, microbiologically confirmed cases and for younger patients, while there was no significant correlation between underreporting rate and nationality.

**Table 1:** Main characteristics of the patients with a “confirmed” diagnosis of TB, according to the notification or not of TB diagnosis to the Hygiene Office (IIDS database)

	**Reported TB**	**Not-reported TB**	**p**
No.	213	80	
Age, mean (SD)	49.27 (19.9)	54,9 (18.99)	0.032
Sex, F/M	86/127	31/49	0.064
Immigrants/Italians	80/133	27/53	ns
Pulmonary/Extrapulmonary	165/48	49/31	
TB localization			
Cavitary Y/N	67/146	10/70	0.001
Smear: positive/negative	82/131	15/65	0.001
Sputum culture positive: Y/N	131/82	39/41	0.048
MDR pattern Y/N/n.a.	5/161/47	1/47/32	ns
Days in hospital, mean (SD)	31.76 (25.85)	29.31 (25.57)	ns

*n.a*. not available data.

In this sample of CRF, TB was in the first position of the discharge record form in 263 out of 346 patients examined; for these patients, TB diagnosis was confirmed in 246 patients (93%) (Table 2). On the contrary in 83 patients with TB code in the following positions a TB diagnosis was confirmed in 47 patients (56%). Therefore, the PPV of the first position of TB code for a “confirmed TB” diagnosis was 93.5%, while the NPV was 43%.

**Table 2:** Distribution of TB diagnoses according to the position in the HDR, in “confirmed” or “not confirmed” TB cases

	**No.**	**TB code in HDR**	**TB code in HDR**	
		**Primary diagnosis**	**Secondary diagnosis**	
Total	346	263	83	p < 0,005*
Confirmed	293	246	47	
Not confirmed	53	17	36	

*by chi square.

### Second study: TB trend and underreporting rate over time in the TB database B

In the second study, a total of 588 TB diagnoses were obtained from HDR, and 273 from LTR. A certain degree of overlap was found between the two records (195 patients were present in both registers), leading to a total number of 666 cases obtained by these two different registers. In this large TB database, a total of 379 patients were underreported (57%), most of them (329 pts, 87%) were from the HDR register.

Underreporting was significantly greater for Italians than for immigrants (68% vs 18%, p < 0.001%). Underreporting rate was lower in patients with microbiological confirmation *vs* patients without microbiological confirmation (25% *vs*75%, p < 0.001), in patients who had a TB code in the discharge record form in first position *vs* in the following positions (39% *vs* 48%, p <0.001), and was different according to the hospital regimen (hospitalized patients *vs* Day Hospital: 70% *vs* 16%, p < 0.001).

**Figure 2:**
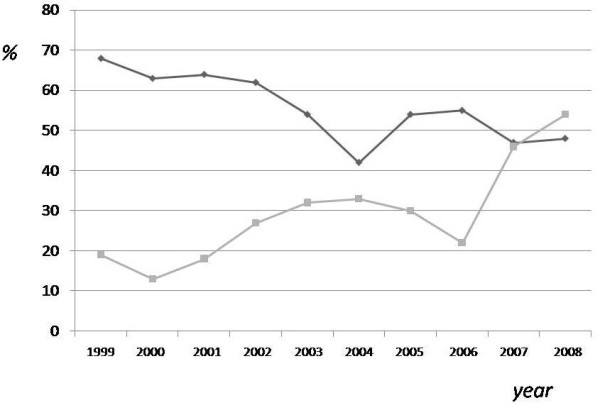
**Underreporting-rate and immigrants rate trend during the study period (1999–2008).** Underreporting rate, in database B patients, during ten years tended to decrease, reaching values of 45% in 2008 (upper line). During the ten years considered, there was a significant increase in the rate of TB diagnosis in immigrants (non Italian cases: 23% in 1999, 62% in 2008) (bottom line).

Underreporting rate progressively decreased over the study period (68% in 1999, 48% in 2008) (Figure 2), probably due to an increasing awareness about TB reporting among clinicians. During ten years, the number of newly diagnosed TB cases in UHP decreased (95 cases in 1999, 42 in 2008), with a significant increase in the percentage of immigrants (non Italian cases: 19% in 1999, 54% in 2008) (Figure 2).

## Discussion

This study confirms that tuberculosis (TB) is still a considerable problem in Central Italy, with almost 700 patients with a confirmed or suspected diagnosis of TB observed in a 10-year period in the UHP. Underreporting TB was substantial, although some decrease was observed in the last years of this survey. However, the accuracy of diagnosis of active TB, as derived from HDR of the three units considered in this study, has been found to be high, with a positive predictive value of 93.5%.

The rate of underreporting we documented in this 10-year period, up to 27% in patients with confirmed TB diagnosis, was unacceptably high. Such failure to notify to the local Hygiene Office of new TB cases may have negative consequences in the epidemiological estimation and hinder the collection of the treatment outcome data. This last one is also affected by the absence of revision of reported TB cases. The notification, in fact, should be sent also for suspected cases, and TB diagnosis should however be excluded at a later time (in our revision 11 cases of “not confirmed TB” were in IIDS register).

As expected, the underreporting rate was higher for in patients treated in Day Hospital and in patients with a TB code in second or following position of the HDR. On the other hand, underreporting was significantly lower for patients with more severe diseases (like patients with cavitary lung lesions) or smear-positive cases. These data are in agreement with similar data collected in other Italian regions or other European countries [12,13,18,19] in the last years, and confirm that the attention towards preventive measures to limit TB diffusion is still lacking.

Several reasons may be taken into consideration to explain such high underreporting rate. Firstly, some Units had not notified their TB cases and, in general, there was poor communication between the different units of UHP and the Hygiene Office as demonstrated by the incomplete overlap among the two different registers. A second reason that may explain why the data are incomplete is the extreme spreading of TB cases across too many different Units (almost 16) in our Hospital. Another point may be the inaccuracy of the TB code reported in the HDR. We were able, indeed, to confirm diagnosis of active TB (by direct examination of the CRF) in 93% of cases with TB code in first position of HDR, in comparison with the 56% only of the cases with TB code in second or following position of the HDR, with a high positive predictive value only for TB code in first position. This represents a limit for the accuracy of this evaluation and suggests that other, more accurate methods for the definition of active TB should be considered.

We considered as “confirmed” TB diagnoses all the cases where the direct examination of the CRF allowed to be confident that the diagnosis of TB was correct (according to the ATS/CDC 1999 criteria) (17). In this case, the TB diagnosis in first position of the HDR suggest that TB was “active” and probably the main reason for the hospitalization.

The number of TB cases observed during this last decade at the UHP was fairly stable over time, with a relative increase in the ratio between immigrants and Italians. The incidence rate cannot be computed, due to the uncertainty of the real full population attending the University Hospital, and because TB patients tend to migrate from one Hospital to the other. However we can speculate that the rate of new TB cases is fairly high (an average of 78 new cases/year was observed).

Therefore, our data have some limitations. Data obtained from some registers (IIDS or LR) may belong to patients treated as outpatients by specialists or GPs or that might have been observed in other hospitals of the large Pisa area. Furthermore, for a considerable number of HDR, no direct examination of the CRF was obtained, and, in addition, accuracy of the TB code was not assessed for this group of patients. Finally, some data derived from other Laboratory Units were missing and not included in the present analysis.

## Conclusion

In conclusion, we confirmed that the rate of underreporting TB is still high in Central Italy in the last 10 years. It is urgent to promote awareness of clinicians and microbiologists of the importance of TB control by implementing a greater centralization in TB management, and by improving communication between different Hospital Units (Mycobacteriology, Medical Direction, Medical Units, Preventive Services).

## Abbreviations

CRF: Clinical record forms; HDR, Hospital Discharge Records; IIDS: Italian Infectious Disease Surveillance; LR: Laboratory Register; MT: Mycobacterium tuberculosis ; TB: Tuberculosis; UHP: University Hospital in Pisa.

## Competing interests

The Authors declare they do not have any conflict of interest nor affiliation with any organization whose financial interest may be affected by material in the manuscript, or which may potentially bias it.

## Authors’ contributions

LM conceived the study, participated in its design and wrote the manuscript. UV and FM acquired data on patients admitted to the Infectious Disease Unit and participated in their analysis and interpretation. FLD and FN acquired data on patients admitted to the Respiratory Pathophysiology Unit and participated in their analysis and interpretation. MC and MG acquired data from the archives of the University Hospital of Pisa and participated in their analysis and interpretation. LG acquired data on patients admitted to the Pneumology Unit and participated in their analysis and interpretation. FA performed data linkage and statistical analysis. LR acquired data on patients admitted to the Mycobacteriology Unit and participated in their analysis and interpretation. PLP and GF made contributions to conception and design, to the analysis and interpretation of data, was involved in drafting the manuscript, and revised the final version. All authors read and approved the final manuscript.

## Pre-publication history

The pre-publication history for this paper can be accessed here:

http://www.biomedcentral.com/1471-2458/12/472/prepub

## References

[B1] Migliori GB Cirillo DM Spanevello A Codecasa LR Stop TB Italia group Ripped from the headlines: how can we harness communication to control TB? Eur Respir J 2007 30 194 198 10.1183/09031936.00069707 17666555

[B2] Storla DG Yimer S Bjune GA A systematic review of delay in the diagnosis and treatment of tuberculosis BMC Public Health 2008 8 15 23 10.1186/1471-2458-8-15 18194573 PMC2265684

[B3] Maclaren Wallace R Kammere JS Iademarco MF Althomsons SP Winston CA Navin TR Increasing proportions of advanced pulmonary tuberculosis reported in the United States : are delays of diagnosis on the rise? Am J Respir Crit Care Med 2009 180 1016 1022 10.1164/rccm.200901-0059OC 19679694

[B4] Raviglione MC Rieder HL Styblo K Khomenko AG Esteves K Kochi A Tuberculosis trends in Eastern Europe and the former USSR Tuber Lung Dis 1994 75 100 416 10.1016/0962-8479(94)90113-9 7718828

[B5] Raviglione MC Sudre P Rieder HL Spinaci S Kochi A Secular trends of tuberculosis in Western Europe Bull World Health Organ 1993 71 297 306 8324847 PMC2393496

[B6] Migliori GB Spanevello A Ballardini L Neri M Gambarini C Moro ML Trnka L Raviglione MC Validation of the surveillance system for new cases of tuberculosis in a province of northern Italy. Varese Tuberculosis Study Group EurRespir J 1995 8 1252 1258 10.1183/09031936.95.08081252 7489786

[B7] Calpe JL Chiner E Marín J Martínez C López MM Sánchez E Evolucion de la declaracion de la tuberculosis en un area sanitaria de la Comunidad Valenciana desde 1987 hasta 1999 Arche Bronconeumol 2001 37 417 423 10.1016/s0300-2896(01)75111-3 11734122

[B8] Jayshree Pillaye J Clarke A An evaluation of completeness of tuberculosis notification in United Kingdom BMC Public Health 2003 6 3 31 10.1186/1471-2458-3-31 PMC240107 14527348

[B9] Hollo V Zucs P Ködmön C Sandgren A Manissero D Marking 15 years of efforts towards a comprehensive European TB surveillance system: the epidemiological situation of TB in the AEU/EEA in 2009 Euro Surveill 2011 16 12 pii:19822 21457687

[B10] World Health Organization Report 2010 http://www.who.int/tb/publications/global_report/en/index.html

[B11] Buiatti E Acciai S Ragni P Tortoli E Barbieri A Cravedi B Santini MG The quantification of tuberculosis disease in an Italian area and the estimation of underreporting by means of record linkage Epidemiol Prev 1998 22 237 241 10052262

[B12] Baussano I Bugiani M Gregori D van Hest R Borracino A Raso R Merletti F Undetected burden of tuberculosis in a low-prevalence area Int J Tuberc Lung Dis 2006 10 415 421 16602406

[B13] Lombardi N Lenzi D Antonioli P Procuranti C Baldi A Levrè E Stima della sottonotifica di Tubercolosi nell’area vasta Nord Occidentale della Regione Toscana, tramite incrocio di più fonti informative Riv Ital Ig 2001 61 456 466

[B14] D.M. 15.12.’90 Sistema informativo della malattie infettive e diffusive (G.U.8.1.1991, n°6)

[B15] D.M. 05.07.’75 Revisione dell’elenco della malattie infettive sottoposte a denuncia obbligatoria (G.U. 29.IX.1975, n°259)

[B16] AUOP (Azienda Ospedaliera Universitaria Pisana) Website http://www.aop.int/ufficiostampa/Anno2009vsAnno2008.pdf

[B17] Diagnostic Standards and Classification of Tuberculosis in Adults and Children This official statement of the American Thoracic Society and the Centers for Disease Control and Prevention was adopted by the ATS Board of Directors, July 1999. This statement was endorsed by the Council of the Infectious Disease Society of America, September 1999 Am J Respir Crit Care Med 2000 161 4 Pt 1 1376 1395 10764337 10.1164/ajrccm.161.4.16141

[B18] Van Hest NA Smit F Baars HW De Vries G De Haas PE Westened PJ Nagelkerke NJ Richardus JH Completeness of notification of tuberculosis in The Netherlands: how reliable is record linkage and capture-recapture analysis ? Epidemiol Infect 2007 135 1021 1029 10.1017/S0950268806007540 17156496 PMC2870642

[B19] Van Hest NA Story A Grant AD Antoine D Crofts JP Watson JM Record-linkage and capture-recapture analysis to estimate the incidence and completeness of reporting of tuberculosis in England 1999–2002 Epidemiol Infect 2008 136 1606 1616 10.1017/S0950268808000496 18346285 PMC2870780

